# The Role of Suicidal Motivations in Foreseeing Suicide Risk in Nonsuicidal Self‐Injury Adolescent Population

**DOI:** 10.1002/cpp.70244

**Published:** 2026-02-25

**Authors:** Alice Wisniewski, Elsa Ronningstam, Marta Moselli, Maria Pia Casini, Riccardo Williams

**Affiliations:** ^1^ Department of Dynamic and Clinical Psychology, and Health Studies, Medicine and Psychology Faculty Sapienza University Rome Italy; ^2^ Harvard Medical School McLean Hospital Belmont Massachusetts USA; ^3^ Department of Human Neuroscience Sapienza University of Rome Rome Italy

**Keywords:** adolescence, emotional regulation, motivations, nonsuicidal self‐injury, psychache, suicidality

## Abstract

Although diagnostically distinct from suicidal behaviour and, by definition, not intended to cause death, nonsuicidal self‐injury (NSSI) remains one of the strongest predictors of suicide. NSSI and suicidal behaviour appear to share a suicidal state of mind, marked by detachment from life and ambivalence toward death. This study investigates suicidal risk in adolescents with NSSI by examining suicidal motivations and regulatory needs linked to psychache and by exploring their contribution to suicidal behaviour. A sample of 134 adolescents with active suicidal ideation, self‐harm or recent suicide attempts was assessed using self‐report measures and structured interviews. Associations between suicidal ideation, motivations and NSSI features were explored using linear regressions. Logistic regressions tested links between suicidal motivations and suicidal attempts in a filtered NSSI subsample. A hierarchical logistic regression compared the associative value of suicidal motivations and personality disorder criteria on suicidal behaviour. Suicidal ideation and motivations were significantly associated with NSSI variables. In the NSSI subsample, interpersonal influence and low fear were linked to suicide attempts. Suicidal motivations showed greater predictive power for suicidal behaviour than personality disorder traits, improving model accuracy. These findings suggest that assessing suicidal motivations in adolescents with NSSI may enhance clinical risk evaluation and support more targeted interventions.

## Summary

- Suicide may serve as a regulatory strategy to manage unbearable psychache.
- In NSSI adolescents, exploring suicidal motivations helps foresee suicidal behaviour.
- Dissociation and aloneness can precipitate suicidal crises in NSSI patients.

## 1 Introduction

Both nonsuicidal self‐injury (NSSI) and suicidality in adolescence and adulthood can play a role in self‐regulation (Boergers et al. 1998; Brown et al. 2002; Nock and Prinstein 2005; Ronningstam et al. 2024; Schechter et al. 2022a; Sprio et al. 2024; Vohs and Baumeister 2002; Wolff et al. 2019). The self‐regulatory function of NSSI and suicidality can be investigated through the analysis of subjective states accompanying these thoughts and behaviours, which translate into specific motivations (Moselli et al. 2021; O'Connor and Pirkis 2016; Schechter et al. 2022a; Toukhy et al. 2024).

It has already been shown that the suicidal mental state contributes to the increase in the intensity of NSSI behaviours (Ferrara et al. 2012; Glenn et al. 2017; Toukhy et al. 2024), but subjective states linked to increased intensity, frequency and severity of NSSI remain underexplored. Furthermore, the most significant regulatory factors in individuals exhibiting such behaviours have not been examined in depth nor have the motivations and regulatory functions of these predominant ideational aspects in NSSI subjects who engage in suicidality.

This work outlines a possible pathway for identifying suicide risk in adolescent patients with NSSI, based on the analysis of suicidal motivations associated with ideation in these patients. The analysis of motivations linked to suicidal ideation in patients with NSSI is proposed here as a useful tool to improve the ability to predict the risk of transition to suicidal behaviour in this population of adolescents.

### 1.1 NSSI: A Brief Overview

NSSI behaviour in adolescents is an ongoing societal health concern, with pooled prevalence rates reported at 17.2% among adolescents, 13.4% among young adults and 5.5% among adults (Swannell et al. 2014).

NSSI refers to an act involving the direct and deliberate destruction of one's body tissue using methods that are socially or culturally sanctioned and without the intent to kill oneself (Nock and Favazza 2009). It should be distinguished from other terms such as ‘self‐injury’, a generic term that also includes suicidal behaviour, and ‘self‐harm’, which refers to intentional, direct and immediate self‐destructive behaviour aimed at damaging the body and which may involve either suicidal intent or not (American Psychiatric Association 2022; Latimer et al. 2013). NSSI typically involves knives, needles, razors or burning with cigarette butts and lighters. The most affected body areas are the front of the thighs and the dorsal side of the forearm (American Psychiatric Association 2022).

Literature has proposed different models of understanding of the psychopathological meaning of NSSI. NSSI has recurrently been described as a mechanism for emotion regulation involved in managing pain, negative or overwhelming feelings and providing temporary relief and sense of control (Chapman et al. 2006; Selby and Joiner 2009; Wolff et al. 2019). In a similar vein, NSSI is reported to allow individuals to regulate dissociative states and feelings of emptiness (Conterio et al. 2015; D'Agostino et al. 2020; Favazza 1996; Kleindienst et al. 2008; Miller et al. 2020). Furthermore, NSSI may act as self‐punishment to cope with intense guilt, inadequacy and unworthiness (Burke et al. 2021; Hooley and Franklin 2018; Rossi Monti 2009; Taylor et al. 2018). Additionally, NSSI can function as a strategy to regulate interpersonal difficulties and relative emotional responses: to communicate distress or elicit reactions from others, to express rage in an indirect way (Ahmed and Stacey 2003; Muehlenkamp et al. 2013; Steggals et al. 2020). In this regard, self‐injurious behaviours may be considered the result of a mind–body dissociation in which the body and the parts of the self that are projected into it are dominated, attacked and eliminated (Brausch and Muehlenkamp 2014; Rossi Monti 2009).

### 1.2 The Relationship Between NSSI and Suicidality

As self‐destructive behaviour, NSSI is commonly distinguished from proper suicidal behaviours based on the intent: NSSI aims to modify, rather than terminate, consciousness. However, suicidal intent is difficult to measure: severe NSSI can have fatal outcomes even without suicidal intent, and suicidal ideation may also occur during nonlethal self‐injury (Latimer et al. 2013).

Furthermore, although diagnostically distinct, NSSI and suicidality demonstrate a significant connection, as evidenced by research findings (Asarnow et al. 2011; Jacobson et al. 2008; Poudel et al. 2022).

In particular, research data indicate that 40%–85% of individuals who engage in NSSI also report a history of suicidal behaviours (Nock et al. 2006; Whitlock and Knox 2007) and that suicidal patients often have a history of NSSI (Jacobson et al. 2008; Langbehn and Pfohl 1993; Nock et al. 2006). In fact, NSSI recurrence is one of the most significant risk factors for suicidal behaviour (Andover and Gibb 2010; Asarnow et al. 2011; Chesin et al. 2017; Duarte et al. 2020; Dulit et al. 1994; Pompili et al. 2015; Victor and Klonsky 2014). Furthermore, research indicates that patients with co‐occurring NSSI exhibit a high level of suicidal ideation, which is a strong predictor of increased frequency and severity of such behaviours, and that many individuals had already contemplated suicide before engaging in self‐harm for the first time (Bryan et al. 2015; Glenn et al. 2017). While the diagnostic distinction remains, evidence suggests a mutual influence between the two phenomena.

The mutual influence between NSSI and suicidal behaviours may be the derivative of the overlap among risk factors increasing the predictability of both conducts (Hamza et al. 2012). Typically, NSSI and suicidal behaviours are both associated with borderline personality disorder (BPD) (Chesney et al. 2014; Muehlenkamp et al. 2010) and depression, childhood traumatic experiences, current life events and PTSD (Adams et al. 1994; Asarnow et al. 2011; Connors 1996; Dougherty et al. 2009; Guertin et al. 2001; Horváth et al. 2020; Jacobson et al. 2008; Kaess et al. 2020; Klonsky and Moyer 2008; Maniglio 2011; Wilkinson et al. 2011; Williams, Chiesa, et al. 2023). Research has also evidenced that the comorbidity of BPD and depression has a cumulative effect, increasing the likelihood of recurrent NSSI episodes as well as persistent suicidal ideation and behaviours (Jacobson et al. 2008). Although NSSI and suicidality are one of the diagnostic criteria for BPD (Moselli et al. 2023), the relationship between BPD, NSSI and suicidal behaviours remains a topic of investigation (Alberdi‐Páramo et al. 2021). Indeed, the rate of BPD patients having attempted suicide in the lifespan is the highest in the population and amounts to 70% percent, with 10% of patients diagnosed with BPD being successful in their attempts (Lak et al. 2025; National Education Alliance for Borderline Personality Disorder 2024). Furthermore, NSSI is a strong risk factor for suicide attempts in BPD patients (March et al. 2007). However, not all BPD patients who engage in NSSI and suicidal ideation (Andrewes et al. 2019) actually hesitate in actual attempts. Identifying factors that differentiate nonsuicidal from suicidal pathways in this clinical population and other adolescents at risk for suicidality may reveal of high preventive value (Ferrara et al. 2012).

Some studies have hypothesized a common process underlying the mutual escalation of NSSI and suicidal ideation and conduct. First, an escalation of NSSI may lead to suicidal attempts when interpersonal distress increases (Gateaway theory; Whitlock and Knox 2007); additionally, NSSI may enhance an individual's capability for self‐harm, as proposed by Joiner (2005). In this regard, Herzog et al. (2022) demonstrated that although NSSI temporarily alleviates suicidal ideation, when repeated, it can lead to an escalation in the risk of future suicidal behaviour (Hamza et al. 2012).

Another line of research has highlighted that both NSSI and suicidality are characterized by a mental state of detachment from life (Ferrara et al. 2012), strong ambivalence toward death (Dickstein et al. 2015; Toukhy et al. 2024) and a profound sense of emptiness (D'Agostino et al. 2020). The strong relationship between recurrent severe NSSI and suicidal outcomes has therefore been attributed to the presence of a common process of emotional regulation as represented by the emergence of the suicidal state of mind.

### 1.3 The Study of the Suicidal Mental State: Core Regulatory Mechanisms and Suicidal Motivations

For a long time, research on suicidality has primarily focused on identifying risk factors, particularly on personality diagnoses and symptomatic comorbidities (Bernal et al. 2007; Chesney et al. 2014; Soloff et al. 2000; Too et al. 2019; Turecki et al. 2019; Yalch et al. 2014). However, based on the current understanding of suicidality, empirical studies have demonstrated that psychiatric diagnoses alone are not sufficient to explain the progression of the suicidal process (Barbagli 2009; Tanzilli et al. 2021).

Within the contemporary understanding of suicidality, which examines the transition from the motivational to the volitional phase of suicide (Klonsky et al. 2016; O'Connor 2011), particular attention has been given to the identification of subjective states that predispose individuals to suicidal crises (Barzilay and Apter 2014; Glenn et al. 2017; Klonsky and May 2014; Maltsberger 2004). Investigating the meaning that suicide holds for the individual becomes essential for predicting its potential outcomes (De Beurs et al. 2019; Schechter et al. 2022a, 2022b). Since the NSSI and suicidality have been conceptualized as stemming from a shared suicidal state of mind (Ferrara et al. 2012; Jacobson et al. 2008; Toukhy et al. 2024), examining these subjective states may provide key insights into the mechanisms driving the mutual escalation of these behaviours.

Within the broader framework of the suicidal process, it has been proposed the role of suicidality as a strategy for self‐regulation (Boergers et al. 1998; Brown et al. 2002; Nock and Prinstein 2005; Ronningstam et al. 2024; Schechter et al. 2022a; Sprio et al. 2024; Vohs and Baumeister 2002; Wolff et al. 2019). In fact, it has been posited that the suicidal process originates from an overwhelming and seemingly unbearable state of psychological and emotional pain—conceptualized as ‘psychache’ (Shneidman 1993)—alongside an urgent need for relief (Schechter et al. 2022a). Such pain can take on different forms and subjective meanings, which in turn shape the significance attributed to NSSI, suicidal ideation and suicidal behaviours, all serving as mechanisms for pain modulation (Ronningstam et al. 2024; Schechter et al. 2022a; Sprio et al. 2024). Therefore, these specific meanings and motivations underlying the self‐destructive behaviours appear to reflect the core personal and interpersonal issues of the individual, which, in turn, form the background of emotional dysregulation. This latter aspect is particularly relevant in adolescence, a developmental phase in which affective instability is frequently observed (Nock et al. 2013). Within this framework, adolescence represents a critical developmental window in which dysregulation‐based processes may emerge and consolidate. Adolescence is indeed the period of onset of both suicidal ideation and conduct, and suicide is considered as the second cause of death in this phase of life in the Western Countries (American Academy of Child and Adolescent Psychiatry 2024). The adolescents' proneness to experience suicidal thoughts and frequent self‐destructive behaviours, including NSSI, is attributed to the background of affective instability (Glenn and Klonsky 2013) due to both the developmental conditions of the brain (Vargas‐Medrano et al. 2020; Williams, Andreassi, et al. 2023) and to the identity, relational and motivational changes (Moehler et al. 2022; Tanzilli et al. 2024) of the period and to an array of contingent experiences including traumatic experiences, family conflicts and victimization (Brent et al. 1993; Waldrop et al. 2007). Since suicide attempts occurring in adolescence are significant predictors of the occurrence of such behaviours in adulthood, focusing on adolescence could be a strong clinical prevention strategy (Nock et al. 2013).

Notably, research has highlighted that the regulation strategies employed in both suicidal ideation and behaviours, as well as in NSSI, manifest through the development of specific suicidal motivations (O'Connor and Pirkis 2016). These motivations direct the suicidal solution and, in turn, facilitate the progression of the suicidal process from ideation toward conduct (Glenn et al. 2017; Klonsky et al. 2016; Orbach et al. 1999; Orri et al. 2014; Shapiro 1970). Therefore, a precise and systematic analysis of the subjective meaning of the suicidal behaviour and NSSI in at‐risk patients may be achieved by accurately identifying their underlying motivations (Moselli et al. 2021; Toukhy et al. 2024). A comprehensive knowledge of these motivations can provide a significant clinical contribution (Casini et al. 2024), especially in patients who struggle to understand and express their suicidal intentions, which are often implicit or even dissociated (Briggs et al. 2019; Brüdern et al. 2024; Hallford et al. 2023; Millner et al. 2017; O'Connor and Pirkis 2016; Schechter et al. 2022a; Schuck et al. 2019). Given the relevance of the partial overlap of suicidal process and NSSI, and in light of the evidence that both suicidal ideation and NSSI represent the strongest predictors of suicide attempts, the isolation of suicidal motivations appears particularly suitable in enhancing the understanding of the suicidal risk in individuals presenting with NSSI.

The present study aims to achieve the following objectives:

- Test whether and to what extent the intensity of suicidal ideation is associated with the duration, frequency and severity of NSSI.
- Identify motivations and regulatory needs linked to NSSI and their association with NSSI clinical features.
- Identify which motivations and regulatory needs are most strongly associated with attempted suicide in subjects with co‐occurring NSSI.
- Identify the relative contribution of motivations and regulatory needs and personality variables in predicting engagement in suicidal behaviours in subjects with co‐occurring NSSI.

## 2 Material and Methods

### 2.1 Sample Selection and Study Design

The sample consisted of 144 adolescents, aged between 12 and 18 years, recruited between 2017 and the early months of 2020, at a metropolitan Italian Paediatric Hospital, in the mood outpatient clinic and in the inpatient ward of the child and adolescent neuropsychiatry unit. The sample had been referred for suicidal ideation and suicidal behaviours or NSSI. Subjects with intellectual disabilities (IQ < 70; *N* = 2), severe impairments in adaptive and school functioning (*N* = 3) or a diagnosis of Autism Spectrum Disorder according to DSM‐5 (*N* = 5) were excluded from the study. The remaining 136 subjects were assessed using a combination of anamnestic and diagnostic self‐report measures and semistructured interviews. Active suicidal ideation was confirmed if the Columbia Suicide Severity Rating Scale (C‐SSRS) score was ≥ 2 for the severity of suicidal ideation.

Independent research psychologists were trained to meet reliability criteria. Each rater participated in regular supervision meetings with a senior psychiatrist experienced in the study's instruments. Coding and data entry were consistently monitored. Each rater was responsible for administering and scoring only one of the measures for the sample and was blind to the evaluations from the other measures.

All patients included in the study were regularly treated and monitored for 6 months following admission. Specifically, clinical monitoring for relapse of suicide attempts was conducted during this 6‐month observation period. Notably, patients who reported suicidal ideation, but no suicide attempts at admission, did not attempt suicide during the 6‐month follow‐up (this subgroup was considered as ideators only). Only two patients who had attempted suicide at admission were reported to have another suicide attempt during the 6‐month follow‐up, with one experiencing a single episode and the other experiencing two episodes. When reassessed with the C‐SSRS, these subsequent attempts showed a lower level of potential lethality compared to the initial assessments at admission. The variable ‘number of suicide attempts’ included in the study refers to the total count of episodes from 3 months prior to admission through the 6‐month follow‐up period.

For specific analyses targeting the relationship between suicidal ideation and NSSI behaviours, a filtered subsample of 100 subjects who engaged in NSSI was used. This subsample was selected to focus exclusively on individuals presenting NSSI, ensuring a targeted investigation of its motivational and behavioural aspects. Of these, 43 patients were classified as Ideators and 57 as Attempters.

### 2.2 Measures

General cognitive functioning was assessed through scaled tests based on age and language, including the Raven Progressive Matrices Test (Raven 1981) and the Wechsler Intelligence Scale for Children‐Revised (WISC‐IV; Orsini et al. 2021). The subjects' intellectual abilities were classified according to the Diagnostic and Statistical Manual of Mental Disorders, 2000 (DSM‐IV‐TR).

*The Columbia Suicide Severity Rating Scale*; Columbia University (C‐SSRS; Posner et al. 2011) is a scale that evaluate suicidal ideation in subjects aged 12 and over. The scale assesses the severity of suicidality in the domains of suicidal ideation and suicidal behaviour. The C‐SSRS rates four constructs: (a) The severity of the suicidal ideation, measured on a 5 points Likert scale (1, *Desire to be dead*; 2, *Non‐specific active suicidal thoughts*; 3, *Suicidal thoughts with a method*; 4, *Suicidal intent*; 5, *Suicide intent with a plan*); (b) the intensity of the suicidal ideation is reckoned by investigating frequency, duration, degree of control, deterrents and reasons for the ideation; (c) suicidal behaviour rated for actual attempts, aborted attempts, preparatory acts and NSSI; and (d) the lethality of the behaviour. The C‐SSRS psychometric properties, validity and satisfactory internal consistency (Cronbach's alpha = 0.937) have been published (Posner et al. 2011). The scores were obtained after the administration of the specific semistructured clinical interview. The assessment of variables related to NSSI, such as duration, modality, lethality and frequency, was conducted through an anamnestic interview.

*Schedule for Affective Disorders and Schizophrenia for School Age Children, Present and Lifetime* (K‐SADS‐PL, Kaufman and Schweder 2004) is a semistructured interview that was used to assess current and past psychopathology and psychiatric disorders in children and adolescents according to the *Diagnostic and Statistical Manual of Mental Disorders, Fifth Edition (DSM‐5*, American Psychiatric Association 2013) criteria. All patients and at least one parent or guardian were interviewed. This interview was used to identify the presence of MDD.

*Structured Clinical Interview for DSM‐5 Personality Disorders* (SCID‐5‐PD, First et al. 2015) is a semistructured interview that assesses the presence/absence of the 10 Personality Disorders according to DSM‐5 criteria. The average number of PDs per patient and the total number of individual positive traits were then calculated based on the number of criteria met at SCID‐5‐PD. The Italian version of SCID‐5 has good psychometric features: intraclass correlation coefficient (ICC) values ranged from 0.88 (Dependent PD and Histrionic PD) to 0.94 (Avoidant PD) for dimensional SCID‐II‐ interview dimensional ratings (median ICC value = 0.94). Cohen *k*‐values were also adequate for SCID‐II interview categorical PD diagnoses (median *k*‐value = 0.89, SD = 0.11) (Somma et al. 2016). The presence of a PD diagnosis was scored when the subject received at least one categorical diagnosis of the 10 PDs. The dimensional scores for each PD were obtained by summing the number of criteria met by each subject for any of the 10 PDs. The variable PD dimensional overall was obtained by summing all the PD criteria per subject.

*Motivational Interview for suicidality* (MIS‐A, Moselli et al. 2021) is a semistructured clinician‐report interview developed to assess adolescents' motivations underlying the suicidal process. It consists of 11 questions grouped into three sections. These sections encompass questions about suicidal ideation, suicide intention and suicide attempts. (Somma et al. 2016). The MIS‐A distinguishes seven motivational areas and several relative subareas: (a) the ‘illness‐motivated attempts’(including the subareas: psychache and hopelessness), which is associated with unsustainable pain and feelings of powerlessness; (b) the ‘feelings of vulnerability and self‐devaluation caused by chronic pessimistic self‐criticism’ (including the subareas: pessimism and perfectionism), which concerns motivations related to self‐devaluating thoughts and compensatory perfectionism, leading to chronic feelings of shame, guilt and frustration, (c) the ‘life crisis that threatens the cohesion of identity and personal status’, which is, external events that trigger the experience of worthlessness, loss of personal value and failure; (d) the ‘relational’ area (including the subareas: interpersonal influence, closeness seeking, burdensomeness and low belongingness), which refers to motivations that directly call the other into question, as a specific cause of internal pain, or in attempt to affect others and change a relationship; (e) the ‘sense of defeat and entrapment’ (including the subareas: escape fantasy and problem‐solving), related to feelings of having lost every strategy to cope with distress and being trapped into personal vulnerability with no way out; (f) the ‘extreme cases’ (including the subareas: atypical and extreme), which includes both unusual reasons (e.g., psychotic thoughts and gender incongruence) for suicide and the extreme cases where pertaining pastor recent traumatic experiences; and (g) the area of ‘action’ (including the subareas: impulsivity, low fear and no anticipation of pain) describes the mentalization of the consequences of suicide. MIS‐A showed good psychometric properties (e.g., Moselli et al. 2021). Two expert independent judges evaluated MIS‐A with interrater reliability (Cohen's *K*) of 0.71.

### 2.3 Statistical Analysis

Statistical analyses were conducted using Jamovi version 2.3.28. Descriptive analyses were performed to examine the distribution of sex, psychiatric and personality disorders within the sample. In addition, the frequencies of NSSI variables were extracted, and ideators were distinguished from attempters. Nonparametric bivariate correlations were used to test the significance of the associations between self‐harm variables (duration, severity and frequency) and the intensity of suicidal ideation and suicidal motivations. To investigate predictors of self‐harm variables, the general linear model was used. Specifically, a regression model was created for suicidal ideation intensity with NSSI frequency as the dependent variable. For suicidal motivations, three models were developed, with the dependent variables being the duration, frequency and gravity of NSSI. This approach allowed us to explore the distinct contributions of suicidal intensity and motivations across these three dimensions of self‐harm. Finally, to determine which motivations were most strongly associated with suicide attempts in NSSI patients, a correlation analysis was initially conducted to identify suicidal motivations significantly associated with suicidal behaviour. Subsequently, a logistic regression analysis was performed on a selected NSSI sample, with suicidal behaviour as the dependent variable and the identified suicidal motivations as independent variables. To investigate the predictive value of suicidal motivations compared to personality disorder criteria in explaining suicidal behaviours, a hierarchical logistic regression was run. In the first step, personality disorder entered as a predictor of the dependent variable (suicidal behaviour). In the second step, suicidal motivations were added to the model. The improvement in predictive power was evaluated by comparing the changes in model fit statistics (*χ* ^2^ difference test).

## 3 Results

Descriptive analyses revealed that the sample comprised 107 females (78.7%) and 29 males (21.3%). Regarding psychiatric diagnoses, 73 participants (53.7%) met the criteria for MDD, 52 (38.2%) for anxiety disorders, 31 (22.8%) for substance use disorders and 14 (10.3%) for eating disorders. The criteria for the categorical diagnosis of personality disorders were met by 31 subjects (24.8%). In total, the sample included 22 diagnoses of BPD, eight of Avoidant Personality Disorder (APD), three of Narcissistic Personality Disorder (NPD), one of Histrionic Personality Disorder (HPD), one of Paranoid Personality Disorder (PPD) and one of Dependent Personality Disorder (DPD). Some patients presented some of these diagnoses in comorbidity, specifically: One patient had both BPD and PPD; one was diagnosed with APD, DPD and NPD; and one had both HPD and BPD.

As the sample had previously been flagged for suicidal ideation and/or behaviours, 134 out of 136 subjects were assessed as suicidal, of whom 60 were ideators and 74 were attempters (Table 1).

**TABLE 1:** Demographic and diagnostic features of the study sample.

Variable	*n*	%
Female	107	78.7
Major depressive disorder	73	21.3
Anxiety disorders	52	38.2
Substance use disorders	31	22.8
Eating disorders	14	10.3
Borderline PD	22	16.5
Avoidant PD	8	6.0
Narcissistic PD	3	2.3
Histrionic PD	1	0.7
Paranoid PD	1	0.7
Dependent PD	1	0.7
Attempters	74	54.4
NSSI subjects	100	73.53
	**Mean**	**SD**
Age	15.8	1.19
PD dimensional	6.85	4.10

*Note:* PD = Personality Disorder; PD dimensional = Number of positive PD criteria met.

Regarding the frequencies related to NSSI variables, the majority of the sample reported a duration ranging from 0 to 11 months (52 subjects) and high severity (39 subjects). In terms of frequency, most of the sample (39 subjects) reported self‐harming behaviour occurring several times a week or daily. These statistics suggest that NSSI tends to be repetitive and methodical (Table 2).

**TABLE 2:** NSSI variables.

Variable	Mean	SD
NSSI duration	15.8	17.7
NSSI frequency	2.03	1.58
NSSI gravity	1.02	0.82

*Note:* NSSI duration (in months); NSSI frequency (1 = isolated episodes; 2 = systematic episodes; 3 = alternating episodic weekly pattern; 4 = from multiple times a week to multiple times a day).

To investigate predictors of self‐harm variables, a correlation analysis was first used to test the significance of the associations between self‐harm variables (duration, severity and frequency) and the intensity of suicidal ideation and suicidal motivations. The correlation matrix revealed significant associations, as shown in Table 3. After that, separate general linear models (GLMs) were conducted for each dependent variable (NSSI duration, frequency and severity).

**TABLE 3:** Correlation with suicidal ideation intensity, suicidal motivations and self‐harm variables.

	Duration self‐harm	Severity self‐harm	Frequency self‐harm
Suicidal ideation intensity	0.153	0.120	0.261 **
Psychache	−0.073	−0.242 *	−0.051
Helplessness	0.053	0.168	0.119
Pessimism	−0.164	−0.221 *	−0.058
Perfectionism	−0.100	−0.131	−0.121
Life crisis	0.013	0.056	0.036
Interpersonal Influence	0.249*	0.271 **	0.172
Help seeking	0.246 **	0.248 *	0.137
Burdensomeness	0.098	0.208 *	0.082
Low belongingness	0.106	0.265 **	0.198 *
Escape	0.110	0.146	0.091
Problem‐solving	−0.109	0.013	−0.027
Extreme cases	0.175	0.136	0.158
Atypical cases	0.059	0.038	−0.025
Impulsivity	0.276 **	0.097	0.139
No fear	0.162	0.194	0.288 **

*Note:* * *p* < 0.05; ** *p* < 0.01.

For NSSI *frequency*, the first model (*R* ^2^ = 0.068) showed that suicidal intensity had a significant impact (*p* = 0.004). The second model, where suicidal motivations were considered as predictors, revealed that *no fear* (*p* = 0.009) was a significant predictor (Table 4A).

**TABLE 4A:** Generalized linear model predicting NSSI frequency.

Predictor variable	Estimate	SE	Beta	*p*
Intercept	2.00	0.14	0.00	< 0.001
Suicidal ideation intensity	0.07	0.03	0.26	0.004
Intercept	2.150	0.153	0.000	< 0.001
Low belongingness	0.190	0.104	0.177	0.071
No fear	0.422	0.159	0.257	0.009

Abbreviation: SE, standard error.

For NSSI *duration*, the model incorporating suicidal motivations highlighted *interpersonal influence* (*p* = 0.035), *closeness seeking* (*p* = 0.050) and *impulsivity* (*p* = 0.019) as significant predictors (Table 4B).

**TABLE 4B:** Generalized linear model predicting NSSI duration.

Predictor variable	Estimate	SE	Beta	*p*
Intercept	17.18	1.83	0.00	< 0. 001
Interpersonal Influence	2.53	1.18	0.216	0.035
Help seeking	2.47	1.24	0.201	0.050
Impulsivity	3.33	1.40	0.240	0.019

Abbreviation: SE, standard error.

Lastly, for NSSI *severity*, the model focusing on suicidal motivations identified *pessimism* (*p* = 0.029), *interpersonal influence* (*p* = 0.033), *closeness seeking* (*p* = 0.045), *burdensomeness* (*p* = 0.008) and *low belongingness* (*p* = 0.033) as significant contributors (Table 4C).

**TABLE 4C:** Generalized Linear Model predicting NSSI Severity.

Predictor variable	Estimate	SE	Beta	*p*
Intercept	1.00	0.75	0.00	<0.001
Psychache	−0.069	0.05	−0.136	0.145
Pessimism	−0.11	0.05	−0.20	0.029
Interpersonal influence	0.11	0.04	0.21	0.033
Help seeking	0.10	0.05	0.19	0.045
Burdensomeness	0.16	0.06	0.25	0.008
Low Belongingness	0.11	0.05	0.21	0.033
No fear	0.06	0.08	0.08	0.41

Abbreviation: SE, standard error.

Subsequently, to identify the suicidal motivations most strongly associated with suicide attempts in NSSI patients, a correlation analysis was first conducted on a selected NSSI sample to explore significant relationships between suicidal motivations and suicidal behaviour (Table 5). This step informed a subsequent logistic regression analysis, on the same sample, which examined the predictive value of these motivations. The regression results indicated that *interpersonal influence* (*p* = 0.017) and *low fear* (*p* = 0.028) were significantly associated with suicide attempts. Additionally, the motivation *escape* approached significance (*p* = 0.063), suggesting a potential contribution to suicidal behaviour (Table 6).

**TABLE 5:** Correlations with suicidal motivations and suicidal behaviour (Pearson's *r*).

	Suicidal behaviour
Psychache	0.033
Helplessness	−0.186
Pessimism	−0.177
Perfectionism	−0.014
Life crisis	0.086
Interpersonal influence	0.336 **
Help seeking	0.156
Burdensomeness	−0.004
Low belongingness	−0.195
Escape	0.237*
Problem‐solving	−0.017
Extreme cases	0.083
Atypical cases	−0.140
Impulsivity	0.172
No fear	0.320 **

*Note:* *p < 0.05; **p < 0.01; Suicidal behaviour, 0 = absence; 1 = presence.

**TABLE 6:** Binomial logistic regression on suicidal behaviour.

Predictor variable	Estimate	SE	*Z*	*p*
Intercept	−0.867	0.402	−2.15	0.031
Interpersonal influence	0.417	0.175	2.39	0.017
Escape	0.323	0.174	1.86	0.063
No fear	0.879	0.399	2.20	0.028

*Note:* Estimates represent the log odds for ‘SUICIDAL BEHAVIOUR = 1’ vs. ‘SUICIDAL BEHAVIOUR = 0’.

Finally, to investigate the predictive value of suicidal motivations compared to personality disorder criteria in explaining suicidal behaviours, a hierarchical logistic regression analysis was conducted. The results revealed that the number of personality disorder criteria was not a significant predictor of suicidal behaviour, either in the first model (*p* = 0.093) or in the second model that included suicidal motivations (*p* = 0.92). Among the suicidal motivations, escape did not significantly predict suicidal behaviour in the second model (*p* = 0.059), whereas interpersonal influence (*p* = 0.025) and fearlessness (*p* = 0.040) emerged as significant predictors. Finally, the addition of suicidal motivations significantly improved the predictive power of the model for suicidal behaviour (*R* ^2^_N_ = 0.0530) compared to the dimensional diagnosis of personality disorder (*R* ^2^_N_ = 0.3087). This was demonstrated by a chi‐square difference of 16.7 (*p* < 0.001), indicating that suicidal motivations contribute additional predictive value beyond personality disorder traits (Table 7).

**TABLE 7:** Hierarchical logistic regression on suicidal behaviour.

Model		Estimate	SE	*Z*	*p*	Δ*R* ^2^	*χ* ^2^
Model 1	Intercept	−0.46	0.54	−0.85	0.393		
	PD_NumbCrit	0.11	0.06	1.68	0.09		
Model 2	Intercept	−0.94	0.66	−1.43	0.151		
	PD_NumbCrit	0.008	0.082	0.100	0.920		
	Interpersonal influence	0.330	0.174	1.889	0.059		
	No fear	0.833	0.406	2.049	0.040		
	Model improvement					0.053	16.7 (*p* < 0.001)

*Note:* Estimates represent the log odds for ‘SUICIDAL BEHAVIOUR = 1’ vs. ‘SUICIDAL BEHAVIOUR = 0’; SE = standard error; PD_NumbCrit = Number of positive PD criteria met.

## 4 Discussion

The present study aimed primarily to investigate the association between the intensity of suicidal ideation and NSSI variables, while simultaneously exploring which areas of regulation and which specific aspects of the suicidal state of mind most characterize NSSI.

As far as the first objective of the study, the results showed that the intensity of suicidal ideation is significantly associated with the frequency of NSSI. These results corroborate previous literature documenting a strong connection between suicidal and self‐injurious phenomena, supporting the hypothesis of a shared suicidal state of mind (Dickstein et al. 2015; Ferrara et al. 2012; Toukhy et al. 2024). From a clinical and preventive point of view, the presence of both suicidal ideation and NSSI in adolescence, as already discussed in the introduction, has been linked to greater BPD severity at age 16 to 19 (Scott et al. 2015). This findings highlight the clinical necessity of preventive intervention targeting emerging patterns of personality disorders in the juvenile population, particular BPD (Chanen and Nicol 2021). Although many early intervention strategies address general psychopathological dimensions of BPD, it is equally important to target specific outcomes, such as self‐destructive behaviours, which are often central in adolescence, and may precipitate the suicidal crises. Indeed certain aspects of subjective experience and regulative strategies typical of BPD appear to constitute a common ground for the precipitation of these crisis (Schechter et al. 2022a; Tanzilli et al. 2021). Taken together, these findings suggest that, in light of the current results on the shared suicidal state of mind, analysing the quality of motivations in BPD adolescents may play an important role in identifying risk trajectories and informing early preventive interventions aimed at reducing progression toward more severe and chronic psychopathology (Pompili et al. 2015).

As for the second objective of the study, the analysis shows that the suicidal motivations most closely associated with NSSI variables span all the subdomains of the relational area of MIS‐A (Moselli et al. 2021), with the addition of fearlessness and impulsivity. The NSSI variables considered are therefore positively associated with two broad regulatory mechanisms, which are expressed through two main motivational categories: one reflecting the communicative significance of the act and the other its dissociative connotation. Specifically, the self‐injurious behaviour can be understood as a powerful communicative tool that presupposes a significant ‘other’, signifying a search for contact and rejection of help (Green 1991; Lingiardi and McWilliams 2020; Ronningstam 2005; Zanarini et al. 2009). In this context, self‐injury appears to express the need to externalize one's pain within a relational dimension. On the other hand, when motivations such as impulsivity and fearlessness are involved, these are associated with a reduced capacity for mentalization and dissociative features, leading to a progressive disconnection from experiences of pain and bodily mutilation induced by self‐harm.

Regarding the third objective, the motivations most significantly associated with suicide attempts in NSSI patients are interpersonal influence and fearlessness. Both motivational areas thus remain present in the findings, but there is a loss of the motivations with a communicative function within the relational area that might indicate a depressive dimension or a connection to reality, probably related to a persistent attachment to life. The sole persisting relational element assumes characteristics of malignant control, reflecting a narcissistic dimension of suicide, which strongly predicts the lethality of the act (Ronningstam 2005), especially in its grandiose dimension (Casini et al. 2024; Williams et al. 2021). Through omnipotence, the narcissistic personality escapes the sense of vulnerability evoked by interpersonal contact, elevating itself above life itself and thereby rendering death less fearsome (Kernberg 1993; Ronningstam et al. 2008; Schechter et al. 2022a; Williams et al. 2024). Alongside interpersonal influence, fearlessness persists, encapsulating the dissociative, nonmentalizing aspect that predisposes individuals to a profound sense of detachment from reality and emotional disconnection from the external world, a condition that significantly heightens suicide risk (Levinger et al. 2016; Orbach 2006; Pompili 2018; Ronningstam et al. 2024).

Overall, these observations highlight the critical role that investigating the subjective state and suicidal motivations could play in suicide risk assessment. This approach may potentially surpass the predictive role of personality disorders, which, while showing significant associations—particularly BPD—with NSSI and suicidality (American Psychiatric Association 2022; Chesney et al. 2014; Swannell et al. 2014), may not fully account for all nuances of risk. The results of hierarchical regression suggest that certain motivations, reflecting specific regulatory needs that are characteristic of the suicidal state of mind, could play a role in precipitating suicidal crises. This effect appears to manifest either independently of other risk factors or as an element that mediates the impact of these factors on the progression of the suicidal process.

From a clinical perspective, these findings suggest that a careful assessment of suicidal motivations may offer a phase‐sensitive and clinically meaningful complement to traditional risk assessment approaches. Beyond the presence of NSSI, suicidal ideation or personality diagnoses, the identification of specific motivational and relational meanings configurations appears crucial in understanding how distress is processed and translated into self‐injurious or suicidal behaviours. In particular, distinguishing between motivations with a predominantly communicative and relational function and those characterized by dissociation, fearlessness and impaired mentalization may help clinicians differentiate patients who remain within a nonsuicidal self‐injurious trajectory from those at heightened risk of escalation toward suicide attempts, and to organize their interventions accordingly. The assessment of suicidal motivations may support clinicians in identifying regulatory needs and subjective states that signal a transition across different stages of the suicidal process (Ronningstam et al. 2024). As clearly evidenced in the clinical and empirical literature, the possibility to identify, share and discuss the suicidal contents with the patients is of the utmost importance in reducing the suicidal risk (Casini et al. 2024), particularly in adolescents with NSSI (Reinhardt et al. 2022). The correct description of the motivations sustaining the suicidal intent may be pivotal in establishing a positive, destigmatizing alliance with the suicidal patient (Tanzilli et al. 2023). Secondly, it may serve as the basis to create a new framework of understanding and tolerance of the emotional experience triggering the suicidal process (Lindner and Schneider 2016). Finally, probing the suicidal sentiment and motivations may reveal critical in making the suicidal ideation itself emerge when it may otherwise proliferate in a dissociative way and dramatically burst in with no previous warning (Schechter et al. 2022a). We would like to emphasize that this approach could be especially valuable in clinical contexts where access to comprehensive longitudinal information is limited, and decision‐making relies heavily on the patient's current mental state.

Among the limitations of this research is the inability to draw causal predictive conclusions due to the cross‐sectional design employed. Additionally, the generalizability of the findings is constrained by both the design and the clinical characteristics and size of the sample. In this regard, although personality pathology—particularly BPD—is known to play a central role in both NSSI and suicidal behaviours, the limited variability of personality disorder diagnoses within the sample, with most cases clustered in BPD, may have constrained the ability to fully capture the contribution of broader personality pathology to suicidality. Moreover, not all participants engaging in NSSI or suicidal behaviours met criteria for BPD, suggesting that the observed patterns are not exclusively driven by borderline pathology. Future studies should therefore focus on samples including a wider range of personality disorder diagnoses.

From a broader methodological perspective, our study exclusively focused on how suicidal process and NSSI intertwine and influence each other, without considering the possibility that these two phenomena might arise from distinct regulatory processes within the same individual. Another limitation stems from the failure to consider the impact of other relevant risk factors, such as mood disorders, post‐traumatic conditions and psychosocial stressors. These aspects pave the way for future developments, such as the adoption of longitudinal designs that allow for more robust predictive conclusions and the inclusion of a greater number of variables to ensure that significant risk factors are not overlooked.

Another necessary enhancement to this research approach on suicidal motivations involves reducing the level of clinician inference in MIS‐A ratings. Developing a questionnaire with items that clearly describe specific instances of the global motivational areas currently encompassed by the MIS‐A could enable productive confirmatory factor analysis. Finally, given the strong prevalence of relational motivations among NSSI patients, exploring emotional reactions of family and clinicians could be insightful. As highlighted earlier, self‐harm may trigger complex relational dynamics, as the ‘other’, despite having a central role, is rendered powerless. This could allow us to explore how these reactions fit into destructive relational dynamics, offering a unique perspective on relationships and its role in influencing suicidal outcomes (Platt and Salter 1987).

## 5 Conclusions

This study highlights the importance of considering suicidal ideation and regulation processes expressed through suicidal motivations as central elements in the understanding and clinical management of individuals with NSSI. The implications of this work suggest that a therapeutic approach that accounts for specific motivations and the emotional state of NSSI patients could be crucial in preventing the progression toward more severe suicidal behaviour. Moreover, given the significant role of interpersonal motivations in NSSI patients, it becomes crucial to examine how these factors develop within the clinical relationship and interact with transferential dynamics (Lindner 2006). This perspective could enhance the ability to effectively manage intolerable emotional states, fostering a stronger therapeutic alliance and ultimately reducing the risk of suicidal behaviours.

## Funding

The authors have nothing to report.

## Ethics Statement

The study was conducted in accordance with the Declaration of Helsinki and received approval from the institutional ethics committee of the Department of Dynamic, Clinical Psychology and Health Studies, Faculty of Medicine and Psychology, University of Rome ‘La Sapienza’ (protocol n. 181, 12th December 2020).

## Consent

Written informed consent was obtained from all participants involved in the study.

## Conflicts of Interest

The authors declare no conflicts of interest.

## Data Availability

The datasets generated and analysed during the current study are not publicly available due to the sensitivity of the matter under investigation but are available from the corresponding author on reasonable request.
